# Empagliflozin effects on iron metabolism as a possible mechanism for improved clinical outcomes in non-diabetic patients with systolic heart failure

**DOI:** 10.1038/s44161-023-00352-5

**Published:** 2023-10-26

**Authors:** Christiane E. Angermann, Carlos G. Santos-Gallego, Juan Antonio Requena-Ibanez, Susanne Sehner, Tanja Zeller, Louisa M. S. Gerhardt, Christoph Maack, Javier Sanz, Stefan Frantz, Valentin Fuster, Georg Ertl, Juan J. Badimon

**Affiliations:** 1 Comprehensive Heart Failure Center, Würzburg University and University Hospital Würzburg, and Department of Medicine 1, University Hospital Würzburg, Würzburg, Germany; 2 Atherothrombosis Research Unit, Icahn School of Medicine at Mount Sinai, New York, NY USA; 3 Mount Sinai Heart, Icahn School of Medicine at Mount Sinai, New York, NY USA; 4 Department of Medical and Surgical Sciences, University of Córdoba, Córdoba, Spain; 5 Institute of Medical Biometry and Epidemiology, University Hospital Hamburg-Eppendorf, Hamburg, Germany; 6 University Center of Cardiovascular Science, University Heart and Vascular Center, University Hospital Hamburg-Eppendorf, and German Center of Cardiovascular Research, Partner Site Hamburg - Kiel - Lübeck, Hamburg, Germany; 7 Department of Stem Cell Biology and Regenerative Medicine, Eli and Edythe Broad Center for Regenerative Medicine and Stem Cell Research, Keck School of Medicine of the University of Southern California, Los Angeles, CA USA; 8 Department of Medicine V, University Medical Centre Mannheim, Mannheim, Germany

**Keywords:** Clinical pharmacology, Predictive markers

## Abstract

Sodium-glucose co-transporter-2 (SGLT2) inhibitors improve clinical outcomes in patients with heart failure (HF), but mechanisms of action are incompletely understood. In the EMPA-TROPISM trial, empagliflozin reversed cardiac remodeling and increased physical capacity in stable non-diabetic patients with systolic HF. Here we explore, post hoc, whether treatment effects in this cohort, comprising patients who had a high prevalence of iron deficiency, were related to iron metabolism. Myocardial iron content estimated by cardiac magnetic resonance T2* quantification increased after initiation of empagliflozin but not placebo (treatment effect: *P* = 0.01). T2* changes significantly correlated with changes in left ventricular volumes, mass and ejection fraction, peak oxygen consumption and 6-minute walking distance; concomitant changes in red blood cell indices were consistent with augmented hematopoiesis. Exploratory causal mediation analysis findings indicated that changes in myocardial iron content after treatment with empagliflozin may be an important mechanism to explain its beneficial clinical effects in patients with HF.

ClinicalTrials.gov: NCT03485222.

## Abstract (web summary)

The results of EMPATROPISM-FE, a post hoc analysis of the EMPA-TROPISM trial performed by Angermann et al., suggest that the beneficial effects of empagliflozin treatment in heart failure populations may be related to changes in iron metabolism.

## Main

Sodium-glucose co-transporter-2 (SGLT2) inhibitors rapidly reduce cardiovascular risk in patients with heart failure (HF)^1–3^, but underlying mechanisms have remained incompletely understood^4,5^.

Among multiple other effects, SGLT2 inhibitors enhance myocardial energetics^5–8^, reverse cardiac remodeling and improve exercise capacity^7,9–13^, irrespective of diabetes status. Several mechanistic trials reported changes in laboratory iron markers, red blood cell (RBC) indices or erythropoietin levels^14–20^, and, more recently, proteomics research revealed complex proteome shifts induced by SGLT2 inhibitors, implicating modifications of iron homeostasis proteins and erythropoietin, among other changes^21,22^. Secondary analyses from clinical trials suggest that SGLT2 inhibitors increase iron mobilization and use and enhance hematopoiesis^17,18,20^. Given the high prevalence and adverse prognostic significance of iron deficiency and anemia in people with HF^23–26^, better clarification of possible biological links between changes in iron status and the beneficial clinical effects of SGLT2 inhibitors is desirable.

Using cardiac magnetic resonance (CMR) imaging, the ‘Are the ‘cardiac benefits’ of empagliflozin independent of its hypoglycemic activity?’ (ATRU-4; EMPA-TROPISM) trial showed that 6 months’ treatment with empagliflozin induced reverse cardiac remodeling and improved exercise capacity^11^. The EMPATROPISM-FE substudy was designed post hoc (1) to determine whether treatment with empagliflozin is associated with changes in myocardial iron content as estimated by CMR-derived T2*; (2) to explore whether changes in myocardial iron content correlate with changes in left ventricular (LV) structure and function and in physical performance that were observed in EMPA-TROPISM^11^; (3) to assess concomitant changes in systemic iron status, RBC indices, hepcidin and erythropoietin; and (4) to identify potential mediators of the LV structural and functional changes.

## Results

### Study patients

Recruitment took place between May 2018 and August 2019. The last patient completed follow-up on 14 February 2020. One patient in the placebo group died, three patients were lost to follow-up and none received iron supplementation, meaning that 80 of the original 84 EMPA-TROPISM participants were eligible for EMPATROPISM-FE.

Patients had a mean age of 62 ± 12 years, and 60% were men. A high proportion of patients from minority ethnic groups was enrolled, and most had ischemic HF etiology. In both study arms, 18% of patients had chronic kidney disease (estimated glomerular filtration rate (eGFR) <60 ml/min/1.73 m^2^), but average eGFR was normal. Baseline characteristics were similar regarding demographic and clinical features, comorbidities, prescription rates of guideline-recommended medical therapy (GRMT) and devices (Table 1), and neither vital parameters nor any of the efficacy outcomes studied in EMPATROPISM-FE differed between study arms (Table 2).

**Table 1:** Patient demographic and clinical characteristics at baseline

Variable	Empagliflozin (*n* = 40)	Placebo (*n* = 40)
Age, years	64 ± 11	60 ± 13
Male sex	25 (63)	23 (57)
Ethnicity		
Caucasian	15 (38)	6 (15)
Hispanic/Latino	18 (45)	22 (55)
African American	7 (18)	9 (23)
Asian	0	3 (8)
HF features and comorbidities		
Ischemic etiology	23 (57)	19 (49)
Hospitalization for HF (last 12 months)	13 (33)	12 (30)
NYHA class I/II	31 (78)	36 (90)
NYHA class III	9 (23)	4 (10)
Hypertension	32 (80)	26 (67)
Atrial fibrillation	8 (20)	7 (18)
CKD	7 (18)	7 (18)
HF therapies		
ACEi/ARB/ARNi	35 (88)	31 (79)
ARNi	19 (48)	14 (36)
Βeta-blocker	34 (85)	36 (92)
Loop diuretic	20 (50)	23 (59)
Thiazide diuretic	3 (8)	1 (3)
MRA	10 (25)	12 (31)
Pacemaker or ICD	6 (15)	4 (10)
CRT	1 (3)	1 (3)

Values are mean ± s.d. or number of patients (%). ARB, angiotensin receptor blocker; ACEi, angiotensin-converting enzyme inhibitor; ARNi, angiotensin receptor neprilysin inhibitor; CKD, chronic kidney disease (eGFR <60 ml/min/1.73 m^2^); CRT, cardiac resynchronization therapy—pacemaker; HF, heart failure; ICD, implantable cardioverter defibrillator; MRA, mineralocorticoid receptor antagonist; NYHA, New York Heart Association.

**Table 2:** Data for efficacy outcome parameters at baseline and observed changes at 6-month follow-up

Variable	Empagliflozin (*n* = 40)		Placebo (*n* = 40)	
	Baseline	6 months	Baseline	6 months
Vital status				
Systolic blood pressure, mmHg	126.5 ± 19.9	116.5 ± 14.5	120.8 ± 18.3	129.8 ± 20.9
Diastolic blood pressure, mmHg	77.3 ± 9.5	72.8 ± 8.9	76.9 ± 12.3	80.2 ± 12.0
Heart rate (ECG), beats per minute	73.7 ± 16.6	67.8 ± 11.2	77.5 ± 14.8	78.8 ± 10.1
CMR imaging				
T2^*^, ms	41.9 ± 4.8	40.5 ± 5.3	41.0 ± 4.9	41.2 ± 5.2
LVEDV, ml	219.8 ± 75.8	194.6 ± 69.7	210.4 ± 68.9	208.9 ± 72.8
LVESV, ml	143.6 ± 66.3	117.0 ± 60.0	135.1 ± 54.8	134.5 ± 58.9
LVEF, %	36.2 ± 8.2	42.2 ± 9.2	36.5 ± 8.0	36.3 ± 8.5
LV mass, g	135.2 ± 45.2	122.0 ± 40.6	131.8 ± 54.4	135.8 ± 61.0
Cardiopulmonary exercise testing				
Peak VO_2_, ml/kg/min	15.3 ± 4.3	16.4 ± 4.4	14.5 ± 3.9	14.0 ± 4.2
6-MWD, m	419.8 ± 93.4	501.3 ± 100.0	451.8 ± 100.6	417.3 ± 110.9
Laboratory markers of iron status and inflammation				
Iron, µmol L^−1^	6.2 (4.8–9.9)	6.6 (4.8–8.4)	7.4 (4.8–8.5)	7.8 (5.3–9.4)
Iron deficiency^a^ (by iron level), *n* (%)	35 (92)	37 (97)	34 (89)	34 (92)
Transferrin, mg dl^−1^	248.8 (233.0–273.5)	259.0 (230.5–293.5)	264.8 (228.5–292.5)	260.5 (214.5–285.5)
TSAT, %	11.2 (7.5–15.8)	10.5 (7.5–14.7)	10.9 (7.1–13.9)	12.0 (9.2–15.3)
Iron deficiency^a^ (by transferrin saturation), *n* (%)	36 (95)	35 (92)	34 (89)	34 (92)
sTfR, ng ml^−1^	1,393 (1,167–1,678)	1,436 (1,266–1,787)	1,522 (1,182–1,789)	1,338 (1,149–1,711)
Ferritin, µg L^−1^	108.1 (144.7–177.3)	74.2 (40.7–134.1)	83.2 (47.5–128.9)	89.9 (54.5–122.6)
Ferritin <100 µg L^−1^ ^b^, *n* (%)	18 (47)	24 (63)	23 (61)	23 (62)
Hepcidin, µg L^−1^	9.3 (4.3–23.5)	6.8 (2.7–13.8)	7.3 (3.7–15.3)	6.7 (4.9–11.2)
hsCRP, mg dl^−1^	1.9 (0.9–4.3)	2.0 (0.8–4.6)	2.7 (1.3–6.3)	2.4 (1.1–7.3)
RBC indices and hematocrit				
Hemoglobin, g dl^−1^	13.2 ± 1.6	13.7 ± 1.8	13.4 ± 1.6	13.5 ± 1.7
Anemia^c^ (by hemoglobin), *n* (%)	13 (33)	9 (23)	11 (28)	8 (20)
Hematocrit, (%)	40.1 ± 4.6	41.7 ± 5.4	41.0 ± 5.0	41.1 ± 5.0
Anemia^c^ (by hematocrit), *n* (%)	11 (28)	8 (20)	9 (23)	8 (20)
RBC count, ×10^12^ per L	4.5 ± 0.5	4.7 ± 0.6	4.6 ± 0.7	4.6 ± 0.6
MCV, fL	89.1 ± 6.2	89.4 ± 6.4	89.0 ± 6.0	90.2 ± 6.0
MCHC, g dl^−1^	33.0 ± 0.6	32.8 ± 0.7	32.8 ± 0.8	33.0 ± 0.7
MCH, pg	29.4 ± 2.3	29.5 ± 2.2	29.2 ± 2.3	29.8 ± 2.2
RBC distribution width, %	14.2 (13.5–15.4)	14.4 (13.8–15.8)	14.4 (13.8–16.4)	14.0 (13.4–15.1)
Erythropoietin				
Erythropoietin concentration, mU ml^−1^	10.1 (7.6–15.7)	10.3 (6.4–15.4)	10.9 (9.0–15.6)	9.5 (6.9–14.5)

Values are mean ± s.d., median (interquartile range) or number of patients (%). ECG, electrocardiogram; IQR, interquartile range; peak VO2, peak oxygen consumption.

^a^Iron deficiency was defined as plasma iron level ≤13 µmol L^−1^ or transferrin saturation <20%.

^b^Ferritin <100 µg L^−1^ also indicates iron deficiency in patients with HF.

^c^Anemia was defined as hemoglobin <13 g dl^−1^ (<12 g dl^−1^) in men (women) or hematocrit <39% (<36%) in men (women).

### Changes in vital status

Empagliflozin reduced systolic and diastolic blood pressure and heart rate (Table 2), with adjusted treatment effects (95% confidence interval (CI) of −14.9 (−22.7, −7.1) mmHg and −7.5 (−11.7, −3.4) mmHg and −9.5 (−13.4, −5.6) beats per minute, respectively).

### Changes in myocardial T2*

**Fig. 1:**
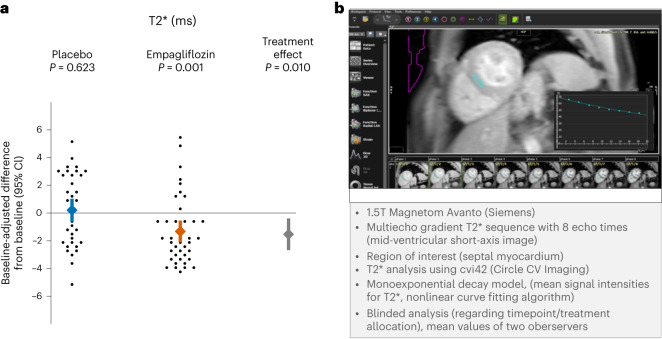
Estimation of myocardial iron content using CMR T2* mapping. **a**, Baseline-adjusted intra-group changes with 95% CI in myocardial T2* from baseline to 6-month follow-up in patients treated with empagliflozin (40 patients) versus placebo (36 patients). Estimated using a baseline-adjusted linear regression model with a two-sided significance level of 0.05, without correction for multiplicity. Results show a significant treatment effect due to a decline of myocardial T2* values in patients treated with empagliflozin. **b**, Mapping technology to quantify parametric T2* as a surrogate marker of myocardial iron content, with lower values suggesting higher myocardial iron content^41^. See text for further details. CV, cardiovascular; ROI, region of interest. Source data

At the 6-month follow-up, T2* had decreased significantly in the empagliflozin group but remained unchanged in the placebo group (adjusted change (95% CI) −1.3 (−2.1, −0.5) ms versus +0.2 (−0.6, 1.0) ms, respectively, and overall treatment effect −1.5 ms (95% CI −2.7, −0.4)) (Fig. 1a).

### Changes in measures of LV remodeling and physical capacity

**Fig. 2:**
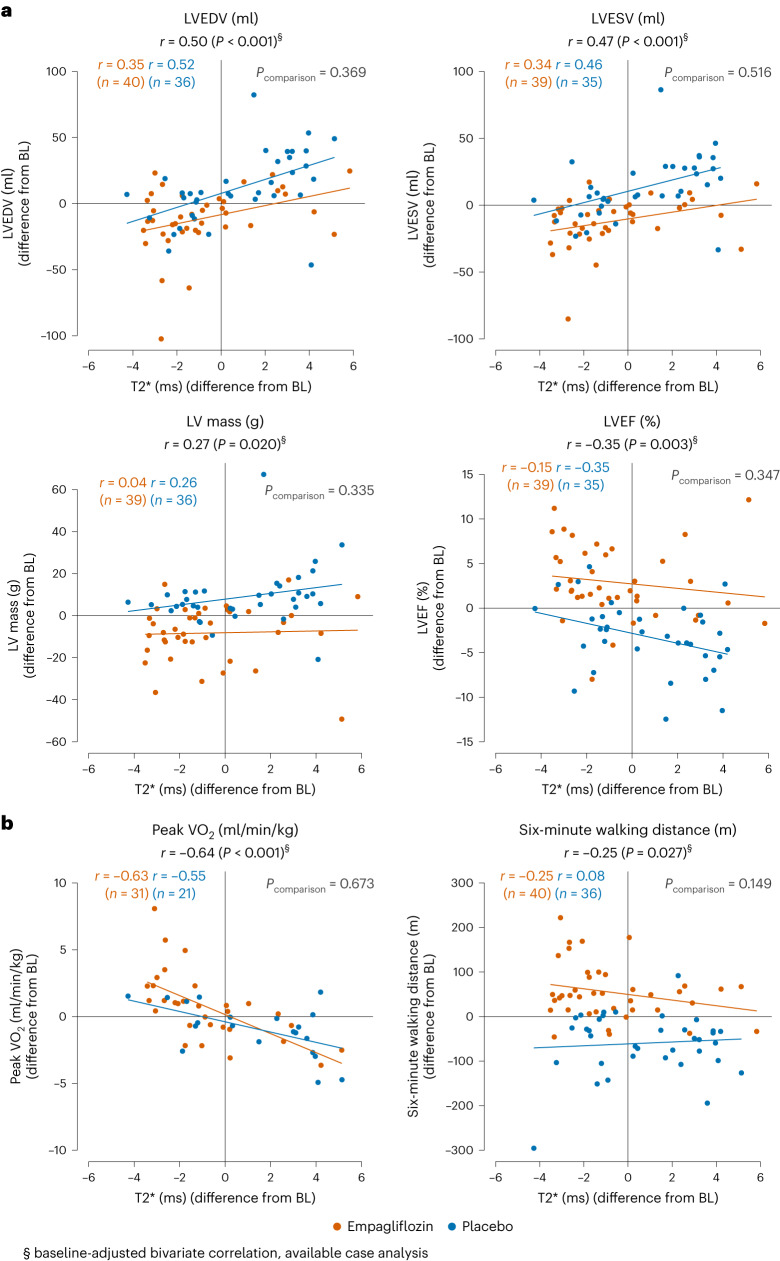
Group-specific correlations and regression lines of changes in T2* and changes in measures of LV remodeling and exercise capacity at 6-month follow-up. **a**,**b**, Group-specific correlations and regression lines of changes in T2* and changes in measures of LV remodeling (**a**) and exercise capacity (**b**) at 6-month follow-up. Pearson correlation coefficients are presented for both study groups and compared for equal correlations using the Jennrich test. In case of a non-significant test, the Pearson correlation coefficient over all patients is given. Results show significant correlations between the changes across study groups. BL, baseline. Source data

At the 6-month follow-up, LV volumes and mass had decreased, and left ventricular ejection fraction (LVEF) had improved in patients receiving empagliflozin but not in patients in the placebo group (Table 2). Baseline-adjusted treatment effects (95% CI) for empagliflozin versus placebo were: left ventricular end-diastolic volume (LVEDV) −23 (−34, −11) ml; left ventricular end-systolic volume (LVESV) −25 (−35, −16) ml; LV mass −17 (−11, −24) g; and LVEF +6.1 (+8.0, +4.2) %. Across study groups, correlations were found between the changes in myocardial T2* and changes in LVEDV (*r* = 0.50), LVESV (*r* = 0.47), LV mass (*r* = 0.27) and LVEF (*r* = −0.35) (Fig. 2a).

After 6 months, exercise capacity had improved in patients on empagliflozin and declined in those on placebo (Table 2). The treatment effect (95% CI) with empagliflozin (*n* = 31) versus placebo (*n* = 22) was +1.7 (0.4, 3.1) ml/kg/min. T2* changes correlated inversely with changes in peak oxygen consumption (VO_2_) across study groups (*r* = −0.64; Fig. 2b, left). The treatment effect (95% CI) for empagliflozin on 6-minute walking distance (6-MWD) was 112 (82, 141) m. Albeit weakly, T2* changes were also inversely correlated with changes in 6-MWD (*r* = −0.25; Fig. 2b, right) across study groups.

### Changes in iron and inflammation markers and in RBC indices

Overall, 69 patients (91%) had a plasma iron level ≤13 µmol L^−1^, and 70 patients (92%) had a transferrin saturation (TSAT) <20% and were, therefore, classified as having iron deficiency^25^. Plasma ferritin was <100 µg L^−1^ in 41 patients (54%). Depending on the definition (hemoglobin <13/<12 g dl^−1^ or hematocrit <39%/<36% in men/women), anemia was present in 24 (30%) or 20 (25%) patients at baseline. Distribution of patients with iron deficiency and/or anemia was similar across study arms (Table 2).

**Fig. 3:**
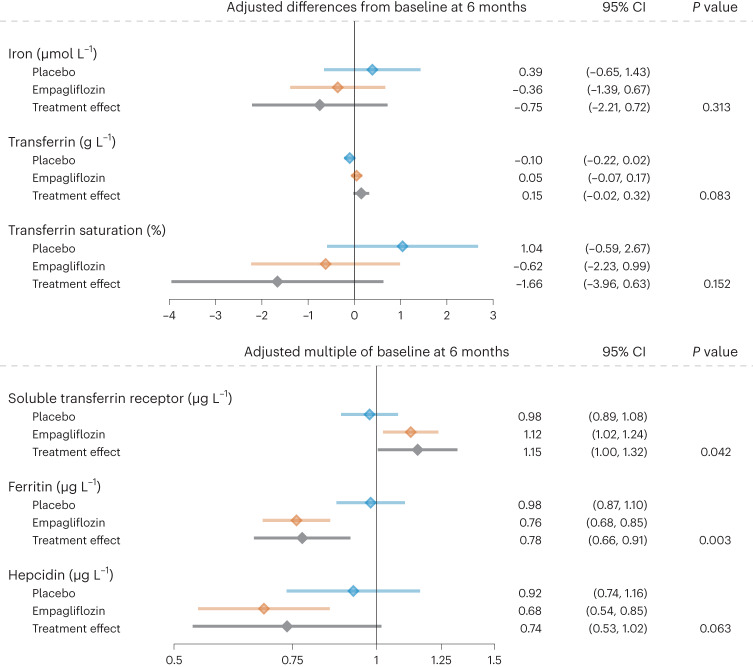
Effect of 6 months’ treatment with empagliflozin versus placebo on laboratory markers of iron status and hepcidin and resulting treatment effects. Data are presented as baseline-adjusted mean differences from baseline with 95% CI, estimated using baseline-adjusted linear regression models for each endpoint with a two-sided significance level of 0.05 and without correction for multiplicity. All analyses are based on data from 80 patients. Source data

In patients receiving empagliflozin, there was a 12% increase in soluble transferrin receptor (sTfR) after 6 months, whereas mean ferritin and hepcidin had declined to 76% and 68% of baseline values, respectively, and iron and transferrin levels, TSAT and high-sensitivity C-reactive protein (hsCRP) were unchanged (Table 2 and Fig. 3). In the placebo group, no changes occurred in any of these markers. Figure 3 shows the treatment effects.

**Fig. 4:**
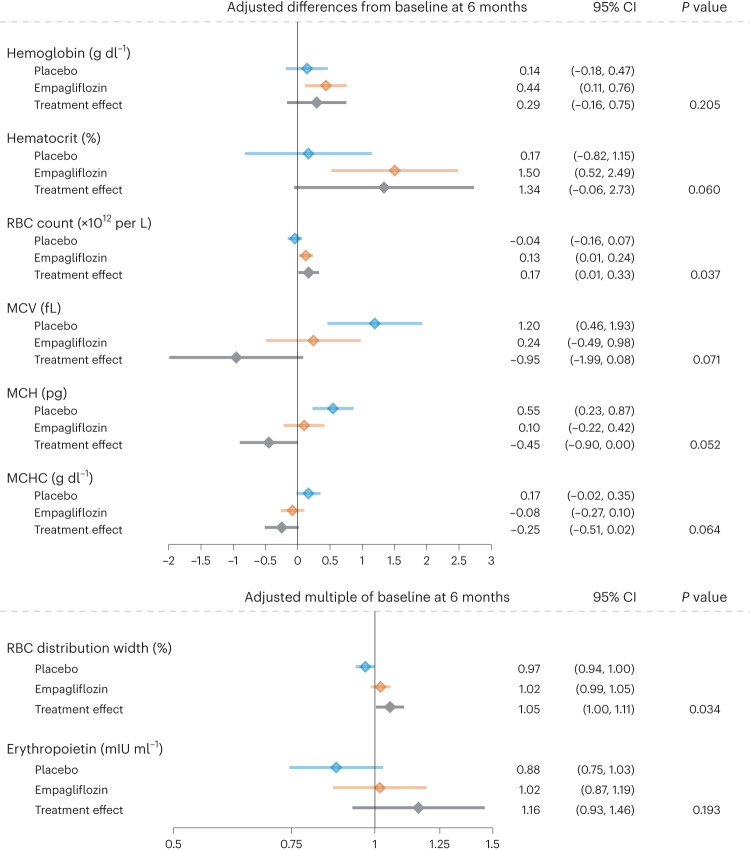
Effects of 6 months’ treatment with empagliflozin versus placebo on RBC indices, hematocrit and erythropoietin and resulting treatment effects. Data are presented as baseline-adjusted mean difference from baseline with 95% CI, estimated using baseline-adjusted linear regression models for each endpoint with a two-sided significance level of 0.05 and without correction for multiplicity. All analyses are based on data from 80 patients. Source data

Six months’ treatment with empagliflozin was associated with increases in hemoglobin, RBC count and hematocrit, whereas these variables were unchanged in the placebo group (Table 2 and Fig. 4). There were no major changes from baseline in mean corpuscular volume (MCV), mean corpuscular hemoglobin (MCH) and mean corpuscular hemoglobin concentration (MCHC) with empagliflozin, whereas all these indices increased in the placebo group (Table 2 and Fig. 4). RBC distribution width tended to increase with empagliflozin and to decrease with placebo. No significant treatment effects on erythropoietin were observed (Table 2 and Fig. 4). Figure 4 shows the treatment effects.

No correlations were found among baseline T2* values, systemic iron markers, hepcidin or hsCRP levels or of changes of these variables after 6 months’ treatment, nor were the changes in T2* related to baseline iron status, hepcidin or hsCRP levels (Extended Data Table 1).

### Causal mediation analysis

**Fig. 5:**
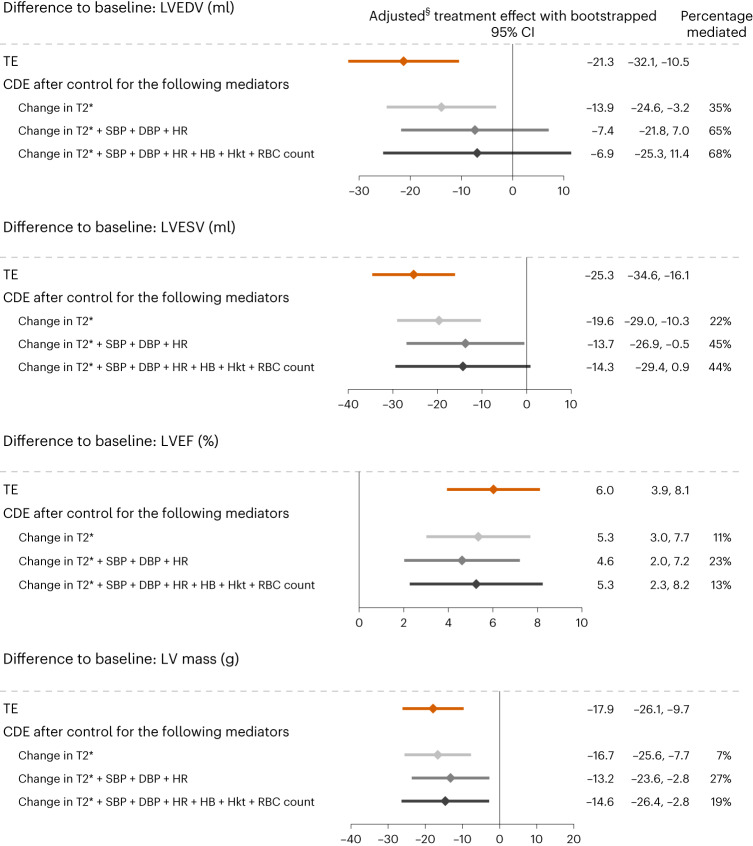
Stepwise multivariable CMAs for the treatment effects on changes in measures of LV structure and function. For each outcome, baseline-adjusted treatment effects with 95% bootstrapped CIs are presented as TE and CDE. The CDE describes the residual treatment effect after controlling for the mediated treatment effect. Shifts in the treatment effect illustrate the contributions mediated with stepwise addition of the three clusters of mediators. All analyses are based on data from 80 patients. DBP, diastolic blood pressure; Hb, hemoglobin; Hkt, hematocrit; HR, heart rate; SBP, systolic blood pressure. Source data

**Fig. 6:**
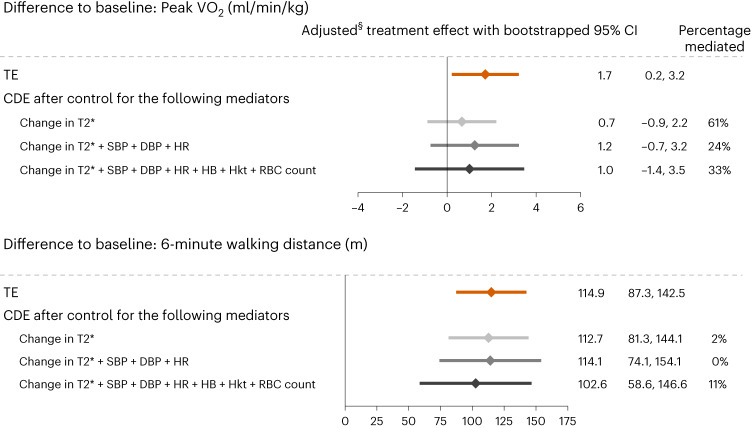
Stepwise multivariable CMAs for the treatment effects on changes in measures of physical capacity. Methods for this analysis are as reported in the Fig. 5 caption. DBP, diastolic blood pressure; Hb, hemoglobin; Hkt, hematocrit; HR, heart rate; SBP, systolic blood pressure. Source data

Depending on the outcome–mediator combinations examined, univariable exploratory causal mediation analysis (CMA) identified changes in T2*, vital parameters and RBC indices as potentially relevant mediators of empagliflozin effects on changes in LV structure and function and changes in exercise capacity, whereas markers of systemic iron status, hepcidin, erythropoietin and hsCRP did not mediate relevant proportions of the empagliflozin effect (Extended Data Table 2). Of these variables, those pertaining to the same mechanistic category were grouped into the following clusters: change in T2*; change in vital parameters (systolic and diastolic blood pressure and heart rate); and change in RBC indices. For each outcome, the adjusted total effects (TE) and the direct effects after controlling for the three clusters of variables were estimated in stepwise multivariable CMA (Figs. 5 and 6). The graphs illustrate that addition of mediators from each cluster shifted the treatment effect toward the zero line. The greater this shift, the higher the proportion of the treatment effect that is explained via the mediator(s) (that is, proportion mediated (PM)) and the smaller the proportion of the treatment effect that would remain if it were possible to eliminate/control for the effect of the mediator(s) on the outcome (controlled direct effect (CDE)).

## Discussion

Key findings from EMPATROPISM-FE are as follows. First, after 6 months’ exposure to empagliflozin, there was a significant treatment effect on CMR-derived myocardial T2* compared to placebo, indicating repletion of myocardial iron content. Although most EMPATROPISM-FE participants had iron deficiency at baseline, treatment with empagliflozin appeared to be associated with further depletion of systemic iron stores. The specific molecular pathways of these changes remain elusive. Beyond the effects of empagliflozin, myocardial iron content is likely to be modulated by other mechanisms implicated in both systemic and intracellular iron homeostasis^27,28^. Second, the magnitude of changes in myocardial iron content correlated with the improvements in measures of cardiac remodeling and physical performance that were previously reported after 6 months’ treatment with empagliflozin^11^. This was consistent across study groups. T2* increases were associated with progressive adverse remodeling. Third, treatment with empagliflozin was associated with changes in various markers of systemic iron status, suggesting increased mobilization and utilization of stored iron^20^. Fourth, changes and trends observed in RBC indices collectively implied augmented hematopoiesis. Finally, exploratory CMA suggested that T2* changes were the most important mediator of the treatment effects observed in EMPA-TROPISM.

Iron is a ubiquitous co-factor for the biogenesis of enzymes, lipids and proteins and for multiple oxidative metabolism processes, erythropoiesis and oxygen transport and storage^27,29^. Experimental data suggest that myocardial iron deficiency leads to progressive cardiac remodeling and hypertrophy and impaired mitochondrial respiration^30,31^. The high prevalence of iron deficiency among people with HF may be related to reduced dietary intake, attenuated absorption due to systemic inflammation and intestinal congestion, drug effects, blood loss or genetic disposition^23,27,29^, but dysregulated iron homeostasis with low TSAT and elevated sTfR levels has also been linked to increased norepinephrine levels^32^. Neurohormonal activation may induce changes in the regulatory molecules of cellular iron homeostasis, resulting in downregulation of mRNA expression of transferrin receptor 1 and inactivation of iron regulatory proteins, thus leading to intracellular iron deficiency and mitochondrial dysfunction^28,33,34^. On the other hand, robust evidence suggests that myocardial iron content is not closely related to systemic iron status^33,35,36^, which may help explain why EMPATROPISM-FE did not, neither at baseline nor during treatment, demonstrate a relationship between myocardial T2*and systemic iron markers and their changes.

A recent randomized trial in iron-deficient patients with HF and reduced LVEF demonstrated that intravenous ferric carboximaltose (FCM) reduced CMR-derived myocardial T2* (ref. ^37^). Although baseline values were similar in both studies, the decrease in myocardial T2* after application of FCM at days 7 and 30 was more than twice that observed in EMPATROPISM-FE. The decrease in T2* induced by FCM correlated with improvements in LVEF at day 30, but 6-MWD remained unchanged^37^. Although T2* decreased more with FCM, functional improvements with empagliflozin were greater and accompanied by reverse remodeling and improved physical performance. Bypassing the tight systemic and cellular homeostatic mechanisms, intravenous iron replacement ameliorates systemic iron deficiency but is unlikely to change the biological mechanisms underlying myocardial iron depletion, such as neurohormonal activation^33,34^. In contrast, our CMA results provide evidence that reduced sympathetic nervous system activity (as suggested by the lower blood pressure and heart rate in EMPATROPISM-FE participants on empagliflozin) may contribute to the beneficial treatment effects on cardiac structure and function and on physical performance.

FCM increases ferritin and TSAT. In contrast, we observed in EMPATROPISM-FE a decline in ferritin, a trend toward lower TSAT and higher transferrin and iron levels. Our results expand on similar findings in non-HF patients with type 2 diabetes treated with SGLT2 inhibitors^14–16^ and are consistent with changes in markers of systemic iron metabolism after 12 months’ exposure to dapagliflozin in a recent analysis from the Dapagliflozin and Prevention of Adverse Outcomes in Heart Failure (DAPA-HF) study^20^. Although the definition of iron deficiency used in the DAPA-HF study is in accordance with guidelines, and therefore has so far remained the ‘official’ definition, it has been questioned because compared with the gold-standard bone marrow staining, other definitions, such as the ones mentioned here, which were also used in EMPATROPISM-FE, did better at diagnosis and at predicting prognosis^25^. The use of different definitions limits comparability of the two studies. Nevertheless, although iron status variables indicated more severe iron deficiency in patients from EMPATROPISM-FE compared to patients from DAPA-HF, treatment-induced changes in systemic iron markers were of similar magnitude in both studies. Together, results suggest that improvements in cellular iron availability and metabolism occurred irrespective of systemic iron status, which may help explain why beneficial clinical effects were observed in both iron-replete and iron-deficient patients in DAPA-HF^20^.

Complementary to findings by Ghanim et al.^15^, who reported an increase in transferrin receptor-1 mRNA expression in patients with diabetes on dapagliflozin, and to observations from DAPA-HF^20^, we found that sTfR levels increased with empagliflozin. The increase co-occurred with rises in hemoglobin, RBC count and hematocrit, changes in other RBC indices and a wider RBC distribution width, all consistent with augmented erythropoiesis.

Mazer et al.^14^ reported similar, albeit more pronounced changes in RBC indices in patients with diabetes after 6 months’ treatment with empagliflozin. More severe iron deficiency, as reflected by substantially lower baseline iron levels and TSAT in EMPATROPISM-FE participants, could have limited their erythropoietic response. Together, these results indicate that increases in hemoglobin and/or hematocrit, as consistently reported from large SGLT2 inhibitor outcome trials^17,18^, likely reflect also augmented erythropoiesis rather than hemoconcentration only.

Previous mediation analysis identified increases in hemoglobin and/or hematocrit as principal mediators of a reduction in clinical outcome events in patients with diabetes and cardiovascular disease treated with empagliflozin^38^, but this analysis did not consider iron metabolism. In EMPATROPISM-FE, an increase in myocardial iron content appeared as the most important mediator of the effects of empagliflozin on EMPA-TROPISM outcomes^14–20^ in exploratory CMA. Interestingly, concomitant changes in RBC indices did not explain relevant proportions of the treatment effect, when added to changes in T2* and vital parameters, although these changes were similar in magnitude to those observed in previous SGLT2 inhibitor trials^14,17–19,38^. Remarkably, CMA also showed that, like changes in hepcidin or hsCRP, changes in markers of systemic iron status appeared unrelated to the treatment effects. This may also explain why beneficial dapagliflozin effects in a large clinical trial occurred irrespective of participants’ systemic iron status^20^.

Several previous studies reported a transient early increase in erythropoietin levels after initiation of an SGLT2 inhibitor, but treatment effects lost statistical significance with time^14–16,19^. Our observation of a non-significant increase in erythropoietin at 6-month follow-up is consistent with this. If anemia is present, erythropoietin increases, to activate hematopoiesis, which, in turn, increases sTfR levels^29,39^. Gradual improvements in RBC indices might, thus, explain the decline of erythropoietin levels with prolonged SGLT2 inhibitor exposure^14,15^.

Similar to Ghanim et al.^15^, who reported that dapagliflozin reduced hepcidin in patients with diabetes, and with findings from DAPA-HF^20^, hepcidin levels tended to decrease with empagliflozin in EMPATROPISM-FE. One possible explanation is depletion of systemic iron stores because hepatic hepcidin production is downregulated with increased iron utilization^29^. However, hepcidin levels are modulated by multiple other factors (for example, inflammation), so the current findings relate to the specific study population but possibly not to patients with different characteristics^23,27,29,31^.

In EMPATROPISM-FE, we demonstrate that treatment with empagliflozin is associated with myocardial iron repletion even in the presence of systemic iron deficiency. Recent proteomics research and evidence from other clinical trials corroborate the relevance of this finding^14,15,20–22^. Although increased iron uptake and utilization in metabolizing tissues after treatment with an SGLT2 inhibitor requires further confirmation, available evidence suggests a potential synergy with therapeutic iron supplementation to replenish deficient iron stores, to further enhance myocardial energetics and to ameliorate anemia. Whether iron uptake by enterocytes is enhanced by SGLT2 inhibitors and whether other organ-specific effects of SGLT2 inhibitors may also be attributable to improved cellular iron availability and use deserve further study.

### Strengths and limitations

This study reports a relationship between changes in myocardial iron content and changes in cardiac remodeling and physical performance after exposure to empagliflozin, and the inclusion of exploratory CMA allowed myocardial iron content to be identified as a potential key mediator of these treatment effects. Another strength of this study is the multi-ethnic nature of the participants. Conversely, the variety of ethnicities represented in this population could limit generalizability due to the impact of inter-ethnic differences in dietary habits and genetic makeup on iron deficiency prevalence and severity^23,40^.

Several other potential limitations should be noted. First, EMPATROPISM-FE was designed post hoc, relying on previously stored images and biomaterials from a relatively small, single-site randomized trial. The modest sample size did not provide sufficient power for analysis of all efficacy outcomes, precluded subgroup analyses and limited exploratory CMA. Furthermore, small sample size and lack of adjustment for multiple comparisons increase the risk of type I error (yet the consistent effects seen in the CMA provide support for the clinical relevance of our findings). Although CMR-derived T2* is currently considered the variable of choice for non-invasive assessment of myocardial tissue iron, it is better established for determining cardiac iron overload rather than assessment of myocardial iron depletion^41^. In addition, some patients had missing cardiopulmonary exercise testing (CPET) assessments, and, therefore, observed changes may not be representative of the entire study population. However, CPET results were consistent with those of the 6-minute walk test (6-MWT), which was performed by all patients. Finally, inclusion and exclusion criteria, especially restriction of the sample to patients with reduced LVEF, limit generalizability.

## Conclusions

Empagliflozin increased myocardial iron content, depleted systemic iron stores and activated hematopoiesis in non-diabetic patients with stable systolic HF and a high prevalence of iron deficiency. Changes in CMR-derived T2* correlated with changes in measures of cardiac structure and function and of physical performance, and the findings of exploratory CMA strengthened the concept that effects of empagliflozin on iron availability and utilization may be an important mechanism to explain its beneficial clinical effects. However, given the modest sample size and post hoc design of EMPATROPISM-FE, our findings should be considered hypothesis generating and need confirmation in a prospective multi-center trial.

## Methods

### Study design

EMPA-TROPISM (NCT03485222) was a single-site, investigator-initiated, double-blind, placebo-controlled trial designed and conducted in accordance with Good Clinical Practice standards. The protocol was approved by the institutional review board of the Icahn School of Medicine at Mount Sinai. Before enrollment, all patients provided written informed consent. Full details of EMPA-TROPISM study design and primary results were previously published^11,42^.

### Study participants

EMPA-TROPISM study inclusion and exclusion criteria were reported previously^11,42^. In brief, male and female outpatients aged ≥18 years with an established diagnosis of HF and LVEF <50% were eligible if they were on stable pharmacological and device therapy for ≥3 months. Key exclusion criteria included any history of diabetes; acute coronary syndrome or cardiac surgery within the last 3 months; eGFR <30 ml/min/1.73 m^2^; systolic blood pressure <90 mmHg; and contraindications to CMR (for example, CMR-incompatible cardiac devices). EMPA-TROPISM participants were eligible for EMPATROPISM-FE if they were completing CMR at the 6-month follow-up and were not receiving any iron supplementation within 6 months before or during the trial.

### Study procedures

After providing informed consent, patients were randomized 1:1 to empagliflozin 10 mg per day or matching placebo added to GDMT using a secure web-based system stratified with block sizes of four^11,42^. Assessments at baseline and after 6 months’ treatment included physical examination, standard laboratory testing, full RBC count, CMR, CPET and 6-MWT. At both timepoints, biomaterials were collected and immediately stored at –80 °C. Interim visits for safety assessments and drug dispensing were scheduled at 1 month and 3 months.

#### CMR imaging

**Extended Data Fig. 1:**
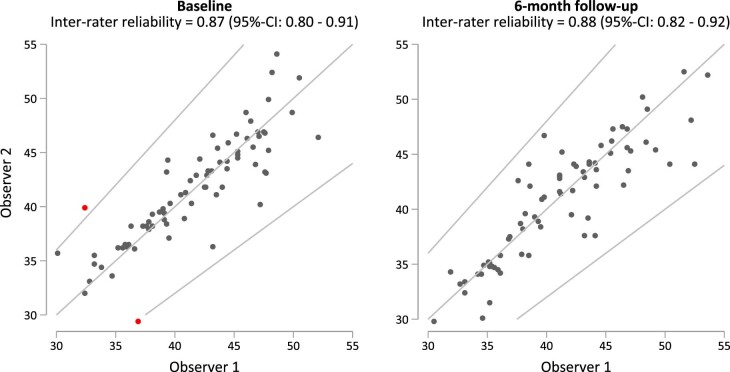
Agreement in assessment of myocardial T2* between two investigators. Shown are interobserver reliabilities of the assessments at baseline (left) and at the 6-month follow-up (right). Each time, the line of identity (y = x) and the range of the 20% deviation of agreement are displayed. Assessments outside this range are shown in red. CI, confidence interval. Source data

CMR was performed on a 1.5-T magnet (Magnetom Avanto FIT, Siemens) using 32-element phased-array coils as receivers. Images were acquired during end-expiratory breathholds and with electrocardiographic gating. Short-axis cine images of both ventricles were obtained from base to apex with a steady-state free precession sequence. In addition, a mid-ventricular short-axis image was acquired using a multi-echo gradient T2* sequence with eight echo times from 2.59 ms to 21 ms. Commercial software (cvi42, Circle Cardiovascular Imaging) was used for image analysis. Epicardial and endocardial contours were traced in each steady-state free precession cine image to obtain LV volumes, LVEF and LV mass^11^. Parametric T2* quantification was performed following recent recommendations for quantitative myocardial tissue iron assessment^41^. A region of interest was placed in the septal myocardium of a mid-ventricular short-axis image, carefully avoiding scar tissue (as determined by late gadolinium enhancement imaging). To quantify T2*, a mono-exponential decay model and nonlinear algorithm were applied for curve fitting of mean signal intensities of increasing echo time images (Fig. 1b). All CMR analyses were performed after study completion. Results are reported as mean values of measurements by two investigators (C.G.S.-G. and J.A.R.-I.) who were blinded to study timepoint and treatment allocation. Reproducibility of T2* measurements was strong (inter-rater reliability (95% CI) at baseline: 0.87 (0.80–0.91) and at 6-month follow-up: 0.88 (0.82–0.92); Extended Data Fig. 1).

#### Exercise testing

To minimize variability, the same sequence of exercise testing was employed at baseline and follow-up, and examinations were supervised by the same personnel. Patients underwent CPET in a fasting state. Upright incremental bicycle ergometry (Lode) with respiratory gas analysis (Med Graphics Ultima O2, MGC Diagnostics) was used. Exercise began with unloaded cycling and increased by 25 W every 3 min. VO_2_, carbon dioxide release (VCO_2_), minute ventilation (VE), perceived level of exertion (Borg scale 6–20), pulse oximetry, heart rate and blood pressure measurements were recorded. Patients were encouraged to exercise until the respiratory exchange ratio was at least 1.1 or the level of perceived exertion was at least 15. The reason for stopping exercise was recorded. Peak VO_2_ was defined as the highest 30-s average of VO_2_. The ventilatory threshold was identified as the point at which the ventilatory equivalent for O_2_ (VE/VO_2_) is minimal, followed by a progressive increase. Ventilation was assessed by correlation of VE and VCO_2_ throughout exercise.

The 6-MWT was performed according to American Thoracic Society guidelines^43^. Patients were instructed to walk as fast and perform as many steps as possible over a 6-min period, without encouragement. Total walking distance was recorded.

#### Laboratory assessments

RBC indices (hemoglobin, MCV, MCH, MCHC and RBC distribution width) and hematocrit were determined at baseline and at 6-month follow-up. Duplicate measurements of markers of systemic iron status (iron, transferrin, TSAT, sTfR and ferritin), inflammation (hsCRP), hepcidin and erythropoietin were performed in the Biomarker Laboratory of the University Heart & Vascular Center Hamburg, using stored EDTA plasma samples. An ARCHITECT c8000 system was used for assessment of iron (Colormetric Multigent Iron Assay, assay range 0.79–179 µmol L^−1^); transferrin (immunoturbidimetric transferrin assay, assay range 0.19–4.77 g L^−1^); and ferritin (Quantia Ferritin assay, assay range 10–500 µg L^−1^), all Abbott Diagnostics. sTfR, hepcidin and erythropoietin were measured with an ELISA: sTfR using the sTfR HS ELISA (assay range 0.145– 160 ng ml^−1^) and hepcidin using the Hepcidin 25 bioactive HS ELISA (assay range 0–81 ng ml^−1^), both DRG. Erythropoietin was measured using the Quantikine IVD ELISA Human Erythropoietin Immunoassay (assay range 2.5–200 mlU ml^−1^), R&D Systems. hsCRP was measured using the immunoturbidometric Multigent CRP vario assay (Abbott Diagnostics) on an ARCHITECT c8000 system (assay range 0.1–160 mg L^−1)^.

**Extended Data Fig. 2:**
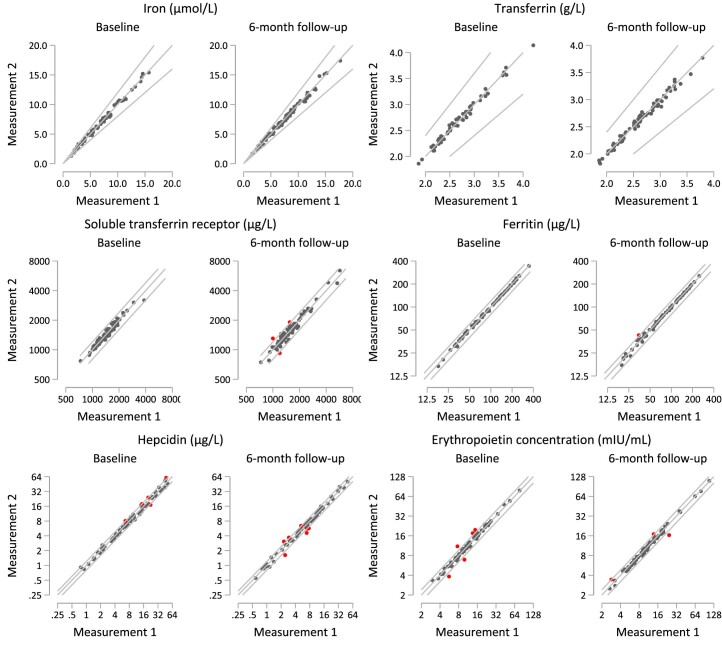
Agreement between duplicate measurements of iron variables, hepcidin and erythropoietin. Shown are intraclass reliabilities of duplicate measurements at baseline (left) and at the 6-month follow-up (right). Each time, the line of identity (y = x) and the range of the 20% deviation of agreement are displayed. Values outside this range are shown in red. The range of intra-class reliabilities is between 0.97 and 1.00. Source data

For quality assurance, the following coefficients of variation (CVs) were determined from the duplicate measurements: iron, inter CV 1.96%, intra CV 1.37%; transferrin, inter CV 0.28%, intra CV 1.08%; sTfR, inter CV 11.94%, intra CV 9.31%; ferritin, inter CV 0.14%, intra CV 1.07%; hepcidin, inter CV 4.86%, intra CV 7.52%; erythropoietin, inter CV 5.19%, intra CV 3.64%; and hsCRP, inter CV 0.72%, intra CV 0.89% (Extended Data Fig. 2). Mean values of the duplicate measurements are reported and used for all analyses.

### Efficacy measures

In EMPA-TROPISM, the primary endpoint was the change in LVEDV between baseline and 6-month follow-up. Secondary outcomes included changes in LVESV, LVEF, LV mass, peak VO_2_ and 6-MWD. The main outcome of EMPATROPISM-FE was change in CMR-derived T2* as a surrogate of myocardial iron content^41^. This was then correlated with the primary and secondary outcome measures from EMPA-TROPISM, followed by CMA. Additional endpoints in EMPATROPISM-FE included changes in markers of systemic iron status (iron, transferrin, TSAT, sTfR and ferritin), inflammation (hsCRP), RBC indices, hepcidin and erythropoietin.

### Sample size and statistical analysis

The number of participants in EMPATROPISM-FE was a consequence of the sample size calculation for EMPA-TROPISM, which was powered for the primary endpoint and included 84 patients^11^.

Continuous variables are reported as mean ± s.d. or median (quartiles) and categorical variables as absolute and relative frequencies. Inter-rater reliability was estimated for repeated T2* measurements (two investigators), and intra-class correlations were determined for repeated laboratory measurements. Continuous variables with skewed distributions were log-transformed.

Treatment effects on T2* (primary outcome), measures of LV structure and function, exercise capacity, systemic iron markers, hsCRP, RBC indices, hepcidin and erythropoietin were estimated using baseline-adjusted linear regression models. Effects are reported as baseline-adjusted difference from, or multiple of, baseline values, as appropriate. Correlations between T2* changes and changes in measures of LV structure and function and, in exercise capacity, were calculated and visualized within study groups. Where within-group correlations did not differ statistically, correlation coefficients across study arms are also reported. These analyses include all patients with available data, with no imputation for missing values.

Exploratory CMA was performed to identify mediators of empagliflozin effects on measures of LV structure and function and on physical performance. CMA aims to decompose the total effect of a treatment (exposure) into a direct effect and a pathway via potential mediator(s) (indirect effect). This pathway is determined by the effect(s) of the treatment on the mediator(s) and the effect of the mediator(s) on the outcome(s), which may depend on the treatment (treatment–mediator interaction)^44^. Potential mediators must be affected by the treatment and be associated with the outcomes of interest. First, the treatment–mediator interaction is considered; if the interaction term does not provide additional explanation regarding the decomposition in the direct effect and the indirect pathway, it can be removed from the model. Subsequently, the following adjusted effects can be estimated:

- TE as an estimate of the treatment effect on the outcome;
- CDE, which corresponds to the direct treatment effect on the outcome after controlling for the treatment effect via the mediator(s);
- The PM (PM = (TE − CDE) / TE), which quantifies to what extent the treatment effect is caused by the mediators.

To estimate and causally interpret these effects, the following three conditions must be met:

(1) no treatment–outcome confounding; (2) no treatment–mediator confounding; and (3) no mediator–outcome confounding.

**Extended Data Fig. 3:**
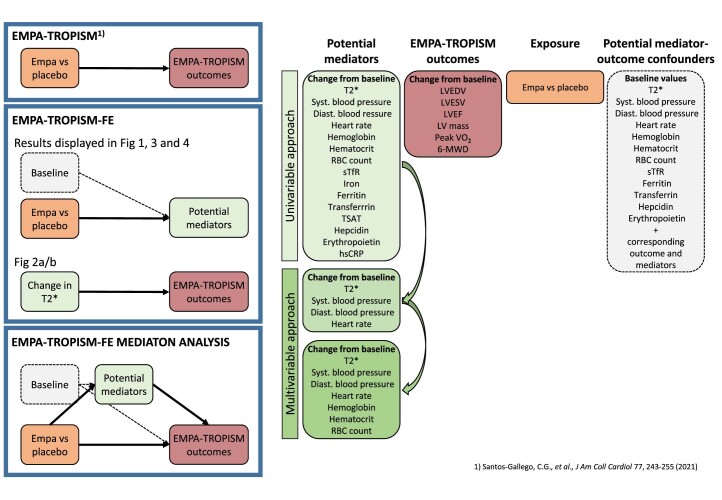
Sequence of analyses applied to EMPA-TROPISM data. Boxes on the left-hand side of the figure show the sequence of analyses performed in the EMPA-TROPISM dataset. In the primary analysis of trial data (top box), the effects of empagliflozin versus placebo on LVEDV, LVESV, LVEF, LV mass, peak VO_2_ and 6-MWD were determined. In the post-hoc EMPATROPISM-FE substudy (middle box), we first evaluated the effects of study treatments (empagliflozin and placebo) on myocardial T2*, vital signs (systolic and diastolic blood pressure and heart rate), laboratory markers of iron status and hepcidin, and indices of red blood cells, hematocrit and erythropoietin. Exploratory mediation analysis (bottom box) was added to determine the proportion of the observed treatment effects that could be attributed to treatment-induced changes in the parameters evaluated in EMPATROPISM-FE. The right part of the figure provides more detailed information on the CMA methodology. CMAs were performed for each of the listed potential mediators and EMPA-TROPISM outcomes. Multiple adjustment of the CMAs was performed by including the listed potential mediator-outcome confounders. LVEDV, left ventricular end-diastolic volume; LVESV, left ventricular end-systolic volume; LVEF, left ventricular ejection fraction; LV mass, left ventricular mass; peak VO_2_, peak oxygen consumption; 6-MWD, 6-minute walking distance; CMA, causal mediation analysis.

In the current study, CMA was performed for each of the following EMPA-TROPISM outcomes: changes from baseline in LVEDV, LVESV, LVEF, LV mass, peak VO_2_ and 6-MWD (Extended Data Fig. 3). The following variables were examined as potential mediators: change from baseline in T2*, systolic and diastolic blood pressure, heart rate, hemoglobin, hematocrit, RBC count, sTFR, iron, ferritin, transferrin, TSAT, hepcidin, erythropoietin and hsCRP. Because the study was a randomized controlled trial, conditions (1) and (2) regarding confounding were fulfilled per se. To fulfil condition (3), multiple adjustments of the CMAs were performed by including the following baseline variables: T2*, systolic and diastolic blood pressure, heart rate, hemoglobin, hematocrit, RBC count, sTFR, ferritin, transferrin, hepcidin and erythropoietin and the examined outcome and potential mediator.

The results of the multivariable CMA are presented in forest plots with bootstrapped 95% CI values with 1,000 replications for the reported effects. In all CMAs, missing values were estimated using the full information maximum likelihood (FIML) procedure^45^.

First, we applied univariable CMA to all potential mediators of each outcome. Subsequently, we grouped variables showing mediating effects on at least one outcome and pertaining to the same mechanistic category into three clusters (T2*, vital parameters and RBC indices) and included those in a stepwise multivariable CMA (Extended Data Fig. 3).

All statistical analyses were conducted at a two-sided significance level of 0.05. Nominal *P* values are reported without correction for multiplicity. Statistical analyses were performed using Stata Statistical Software version 17 (Stata Corp.).

### Reporting summary

Further information on research design is available in the Nature Portfolio Reporting Summary linked to this article.

## Supplementary information

Reporting Summary

## Source data

Source Data Fig. 1 Statistical source data.

Source Data Fig. 2 Statistical source data.

Source Data Fig. 3 Statistical source data.

Source Data Fig. 4 Statistical source data.

Source Data Figs. 5 and 6 Statistical source data.

Source Data Extended Data Fig./Table 1 Statistical source data.

Source Data Extended Data Fig./Table 2 Statistical source data.

## Figures and Tables

**Extended Data Table 1:**
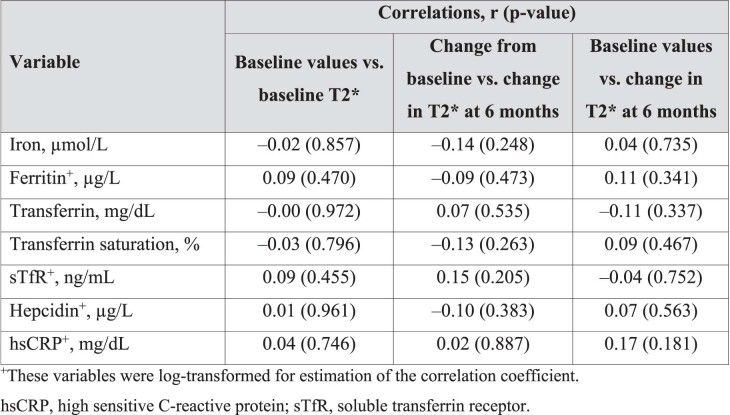
Correlations between T2*values and laboratory iron markers, hepcidin and hsCRP

In the absence of significant treatment group-specific correlations, all correlations (*P* values) are reported across both treatment arms.

**Extended Data Table 2:**
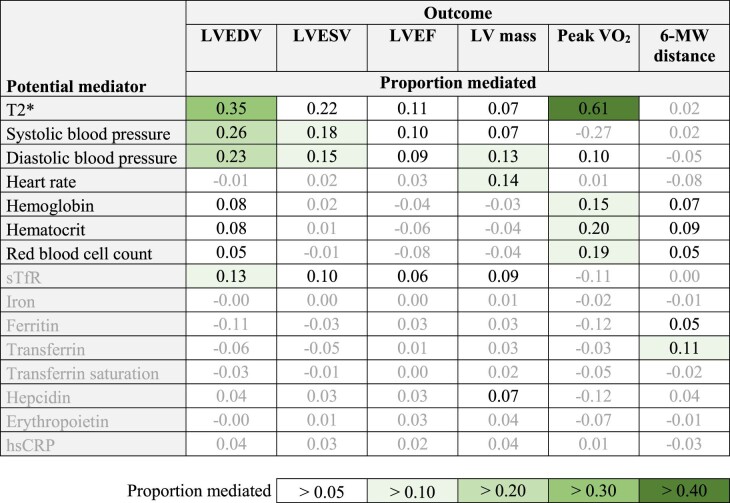
Univariable CMAs for the effect of empagliflozin on changes in measures of LV structure and function and of physical performance (*n* = 80)

Heat map showing proportions mediated in each outcome–mediator combination. The proportions mediated reflect how much of the overall treatment effect on each of the outcome variables is affected by a potential mediator. For each outcome variable and mediator, adjustments for the respective baseline values and for predefined baseline confounders were made.

## Data Availability

De-identified data that support the findings of this study are available from the corresponding author upon reasonable request. Requests will be responded to within 30 working days. Because of the modest sample size and single-center nature of the study, which create a higher potential for re-identification, data will be provided only to qualified researchers with training in human subject confidentiality protocols. Source data are provided with this paper.
