# Supplementary material for: Empagliflozin effects on iron metabolism as a possible mechanism for improved clinical outcomes in non-diabetic patients with systolic heart failure
Source: Nat Cardiovasc Res. 2023 Oct 26;2(11):1032–43. doi: 10.1038/s44161-023-00352-5 (PMC11358002; doi:10.1038/s44161-023-00352-5)
Supplement: Supplementary file 1 — Reporting Summary [file 44161_2023_352_MOESM1_ESM.pdf]

nature portfolio

Corresponding author(s): NCVR-2022-12-1449C

Last updated by author(s): Jul 25, 2023

Reporting Summary

Nature Portfolio wishes to improve the reproducibility of the work that we publish. This form provides structure for consistency and transparency in reporting. For further information on Nature Portfolio policies, see our Editorial Policies and the Editorial Policy Checklist.

Statistics

For all statistical analyses, confirm that the following items are present in the figure legend, table legend, main text, or Methods section.

n/a	Confirmed	
☐	☒	The exact sample size (*n*) for each experimental group/condition, given as a discrete number and unit of measurement
☐	☒	A statement on whether measurements were taken from distinct samples or whether the same sample was measured repeatedly
☐	☒	The statistical test(s) used AND whether they are one- or two-sided<br>*Only common tests should be described solely by name; describe more complex techniques in the Methods section.*
☐	☒	A description of all covariates tested
☐	☒	A description of any assumptions or corrections, such as tests of normality and adjustment for multiple comparisons
☐	☒	A full description of the statistical parameters including central tendency (e.g. means) or other basic estimates (e.g. regression coefficient) AND variation (e.g. standard deviation) or associated estimates of uncertainty (e.g. confidence intervals)
☒	☐	For null hypothesis testing, the test statistic (e.g. *F*, *t*, *r*) with confidence intervals, effect sizes, degrees of freedom and *P* value noted<br>*Give P values as exact values whenever suitable.*
☒	☐	For Bayesian analysis, information on the choice of priors and Markov chain Monte Carlo settings
☒	☐	For hierarchical and complex designs, identification of the appropriate level for tests and full reporting of outcomes
☒	☐	Estimates of effect sizes (e.g. Cohen's *d*, Pearson's *r*), indicating how they were calculated

Our web collection on statistics for biologists contains articles on many of the points above.

Software and code

Policy information about availability of computer code

Data collection	No software was used.
Data analysis	Statistical analyses were conducted using Stata Statistical Software, version 17 (Stata Corp., USA)

For manuscripts utilizing custom algorithms or software that are central to the research but not yet described in published literature, software must be made available to editors and reviewers. We strongly encourage code deposition in a community repository (e.g. GitHub). See the Nature Portfolio guidelines for submitting code & software for further information.

Data

Policy information about availability of data

All manuscripts must include a data availability statement. This statement should provide the following information, where applicable:

- Accession codes, unique identifiers, or web links for publicly available datasets
- A description of any restrictions on data availability
- For clinical datasets or third party data, please ensure that the statement adheres to our policy

Deidentified data that support the findings of this study are available from the corresponding author upon reasonable request. Because of the modest sample size and the single-center nature of the trial, which creates a higher potential for re-identification, data will be provided only to qualified researchers iwith training in human subject confidentiality protocols.

Research involving human participants, their data, or biological material

Policy information about studies with human participants or human data. See also policy information about sex, gender (identity/presentation), and sexual orientation and race, ethnicity and racism.

Reporting on sex and gender	We report sex distribution in the patient sample investigated in this trial ('60% were men'). The moderate size of the study sample did not allow investigation of the principal research question in subgroups according to patient sex.
Reporting on race, ethnicity, or other socially relevant groupings	We report on a study population of elderly non-diabetic patients with sympromatic heart failure and a left ventricular ejection fraction below 50%, who had a mean age of 62±12 years; 60% were men and the majority had ischemic heart failure etiology. A high proportion of the study population were from ethnic minorities.
Population characteristics	Male and female outpatients aged ≥18 years with an established diagnosis of heart failure and a left ventricular ejection fraction below 50% were eligible for the EMPATROPISM study if they had been on stable pharmacological and device therapies for at least 3 months. Key exclusion criteria were: any history of diabetes; acute coronary syndrome or cardiac surgery within the last three months; eGFR <30 mL/min/1.73m2; systolic blood pressure <90 mmHg; contraindications to cardiac magnetic resonance imaging (CMR, e.g. CMR-incompatible cardiac devices). EMPATROPISM participants were eligible for EMPATROPISM-FE if they completed 6-month follow-up including CMR, and did not receive any iron supplementation within 6 months before or during the trial. Baseline patient characteristics were similar in both study arms with respect to demographic and clinical features, comorbidities, prescription rates of guideline recommended therapy, and device therapies. In both study arms, 18% of patients had chronic kidney disease (estimated glomerular filtration rate [eGFR] <60 ml/min/1,73m²), but average eGFR was within the normal range.
Recruitment	Possible candidates were identified from the Consortium Database that includes Mount Sinai Medical Center, Mount Sinai West, Mount Sinai St Luke's, Elmhurst Hospital, and the Bronx VA Medical Center. In addition, patients were recruited by heart failure specialists at cardiovascular clinics and medical centers, and from cardiac rehabilitation programs of the Mount Sinai Health Network.
Ethics oversight	The EMPATROPISM study protocol was approved by the Institutional Review Board of Icahn School of Medicine at Mount Sinai. Before enrolment, all patients provided written informed consent.

Note that full information on the approval of the study protocol must also be provided in the manuscript.

Field-specific reporting

Please select the one below that is the best fit for your research. If you are not sure, read the appropriate sections before making your selection.

☒ Life sciences ☐ Behavioural & social sciences ☐ Ecological, evolutionary & environmental sciences

For a reference copy of the document with all sections, see nature.com/documents/nr-reporting-summary-flat.pdf

Life sciences study design

All studies must disclose on these points even when the disclosure is negative.

Sample size	The number of participants in EMPATROPISM-FE was a consequence of the sample size calculation for EMPATROPISM, which was powered for the primary endpoint (between-groups difference in the change of the LV end-diastolic volume from baseline to 6 months).
Data exclusions	EMPATROPISM participants were eligible for EMPATROPISM-FE if they completed 6-month follow-up including CMR, and did not receive any iron supplementation within 6 months before or during the trial. Since one patient in the placebo group died, three patients were lost to follow-up, and none received iron supplementation, 80 of the original 84 EMPATROPISM study participants were eligible for the EMPATROPISM-FE substudy. No other data than the baseline assessments of these 4 patients were excluded from the analyses.
Replication	All CMR analyses were performed by two independent investigators, who were blinded to study time point and treatment allocation. All results are reported as mean values of both measurements. Assessment of the reproducibility of T2* measurements was particularly critical. In the online supplement we therefore report on the agreement between the two investigators in assessing myocardial T2*. We show correlations of the results of the individual measurements of both investigators at baseline and 6-month follow-up. Correlations between the two investigators were good, with an inter-rater reliability [95% confidence interval (CI)] at baseline of 0.87 [0.80–0.91] and at 6-month follow-up of 0.88 [0.82–0.92]. Duplicate measurements of laboratory markers of iron status, high-sensitive C-reactive protein, hepcidin and erythropoietin were performed after completion of the EMPATROPISM study using stored EDTA plasma samples. Coefficients of variation were calculated for all laboratory markers. Mean values of both measurements are reported. Intra-class correlations were excellent (between 0.97 and 1.00). Thus, in summary, all attempts at replication were successful in this mechanistic study.
Randomization	After providing informed consent, patients were randomized 1:1 to empagliflozin 10 mg/day or matching placebo added to guideline-directed medical therapy using a secure web-based system stratified with block sizes of four.
Blinding	EMPATROPISM was a classical double-blind, placebo-controlled pharmacological trial.

Reporting for specific materials, systems and methods

We require information from authors about some types of materials, experimental systems and methods used in many studies. Here, indicate whether each material, system or method listed is relevant to your study. If you are not sure if a list item applies to your research, read the appropriate section before selecting a response.

Materials & experimental systems

n/a	Involved in the study	
☒	☐	Antibodies
☒	☐	Eukaryotic cell lines
☒	☐	Palaeontology and archaeology
☒	☐	Animals and other organisms
☐	☒	Clinical data
☒	☐	Dual use research of concern
☒	☐	Plants

Methods

n/a	Involved in the study	
☒	☐	ChIP-seq
☒	☐	Flow cytometry
☒	☐	MRI-based neuroimaging

Clinical data

Policy information about clinical studies

All manuscripts should comply with the ICMJE guidelines for publication of clinical research and a completed CONSORT checklist must be included with all submissions.

Clinical trial registration	Trail reg: www.clinicaltrials.gov; NCT03485222
Study protocol	The trial protocol of the EMPATROPISM study has been published (Rationale and Design of the EMPA-TROPISM Trial (ATRU-4): Are the "Cardiac Benefits" of Empagliflozin Independent of its Hypoglycemic Activity? Santos-Gallego CG, Garcia-Ropero A, Mancini D, Pinney SP, Contreras JP, Fergus I, Abascal V, Moreno P, Atallah-Lajam F, Tamler R, Lala A, Sanz J, Fuster V, Badimon JJ. Cardiovasc Drugs Ther. 2019 Feb;33(1):87-95. doi: 10.1007/s10557-018-06850-0.)
Data collection	Clinical data and biomaterials were collected at the Mount Sinai Medical Center, NY, USA in the time period between May 2018, and February 2020, when the last patient completed his trial participation (recruitment took place between May 2018, and August 2019). The biomarker analysis and data collection took place in 2 phases, from April 2021 through July, 2021, and in February, 2022, in the the Biomarker Laboratory of the University Heart & Vascular Center Hamburg, using stored EDTA plasma samples shipped in 2 portions from NY to Hamburg, Germany
Outcomes	The primary endpoint of the EMPATROPISM trial was the between-group difference in the change in left ventricular (LV) end-diastolic volume from baseline to 6 months. The reason for choosing this endpoint was that LV volumes are strong predictors of adverse cardiovascular outcomes even after adjusting for ejection fraction. Cardiac magnetic resonance (CMR) imaging was chosen as the imaging method to assess this endpoint because it is the gold standard for quantifying LV volumes, mass and function; the reproducibility of CMR allows for a smaller sample size compared with other imaging methods, e.g., echocardiography. Secondary endpoints included the between-group differences in changes in peak oxygen consumption, and in 6-minute walking distance, because these variables constitute the gold standard for studying cardiac and pulmonary adaptation to exercise in patients with heart failure. Other endpoints included between-group differences in changes in LV mass and LV ejection fraction, and 6-minute walking distance.<br>Efficacy outcomes of the EMPATROPISM-FE substudy were selected to enable assessment of the specific substudy hypotheses. The main outcome of interest was the between-group difference in the change in CMR-derived myocardial parametric T2* values (as an established surrogate of myocardial iron content) between baseline and the 6-month follow-up. This outcome was then correlated with the primary and secondary outcome measures of the original EMPATROPISM study. Additional substudy endpoints included changes in laboratory markers of iron status (iron and transferrin levels, transferrin saturation, soluble transferrin receptor concentration, ferritin level) and inflammation (hsCRP level), and changes in red blood cell indices, hepcidin and erythropoietin levels. In addition, we performed exploratory causal mediation analysis (CMA) to identify mediators of the empagliflozin effects on measures of LV structure and function and on physical performance. First, univariable CMA was applied to all potential mediators of each outcome. Subsequently, we grouped variables showing mediating effects on at least one outcome and pertaining to the same mechanistic category into three clusters (T2*, vital parameters, RBC indices), and included those in a stepwise multivariable CMA.
